# Supplementary material for: Is Gauchian genotyping of GBA1 variants reliable?
Source: medRxiv. 2023 Oct 26:2023.10.26.23297627. Preprint. [Version 1] doi: 10.1101/2023.10.26.23297627 (PMC10659459; doi:10.1101/2023.10.26.23297627)

Supplemental Figure 1

The complete set of 95 whole genome sequencing samples was processed using Gauchian and the results are presented with the original headers provided by the tool. Sanger-established genotype and gender are provided for the NIH cohort and a final assessment comparing the Gauchian call against the Sanger genotype is presented.

A – Pat_03
Sanger Annotation: N370S/N370S
Gauchian Call: N370S/WT

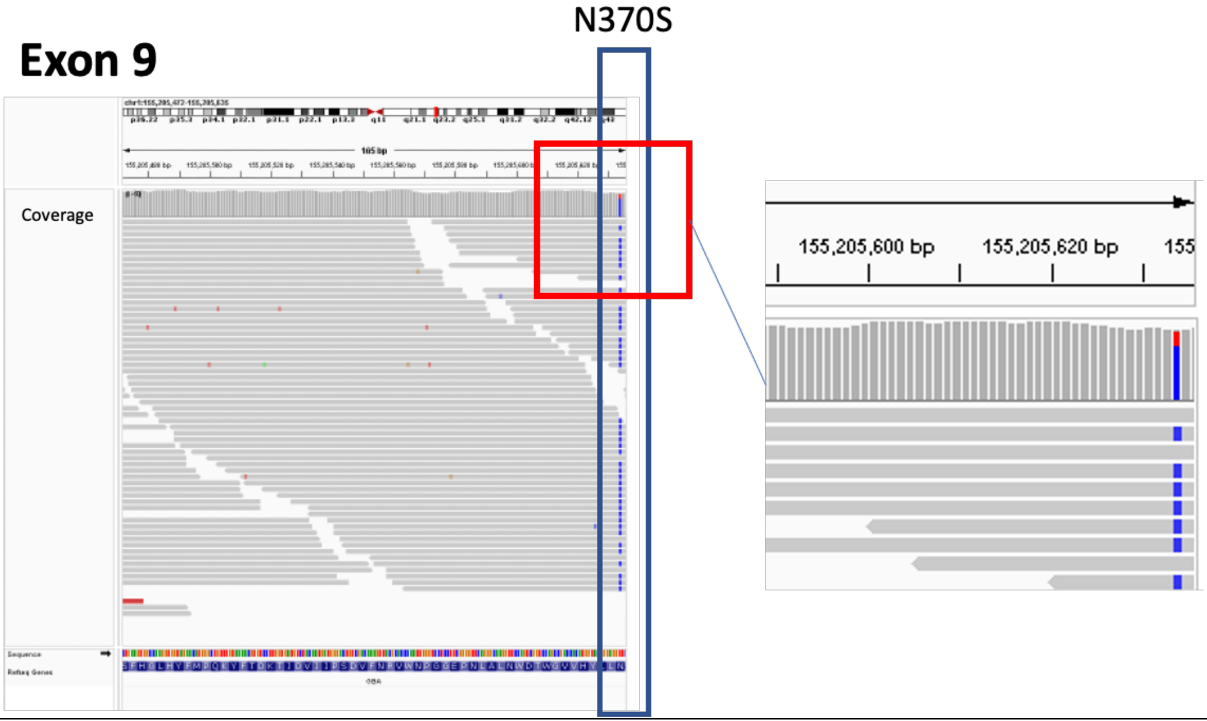

B – Pat_08
Sanger Annotation: N370S/Q350X
Gauchian Call: N370S/WT

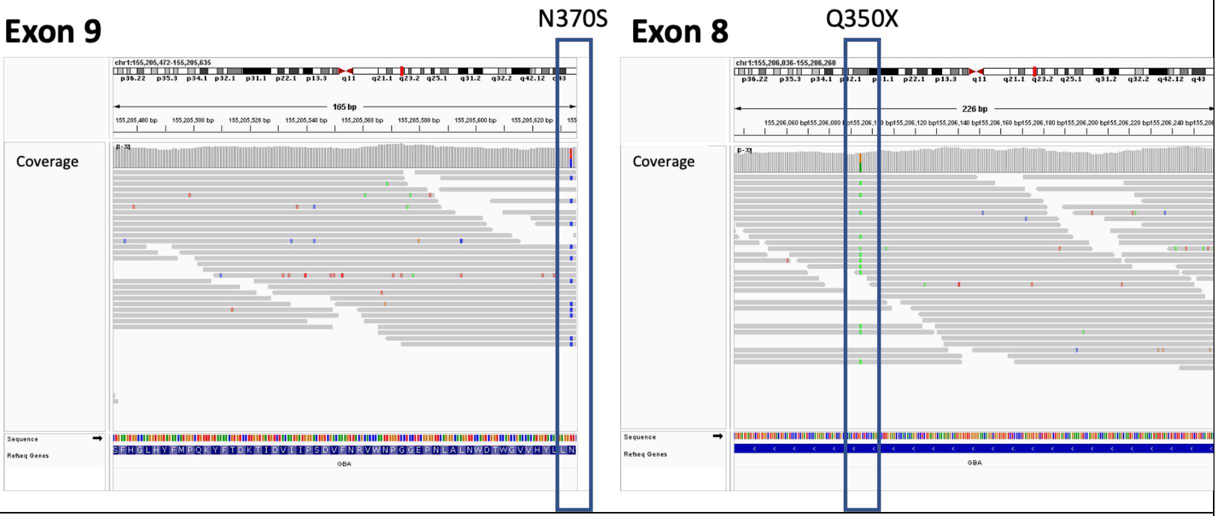

C – Pat_26

Sanger Annotation: N370S/R463C
Gauchian Call: N370S/WT

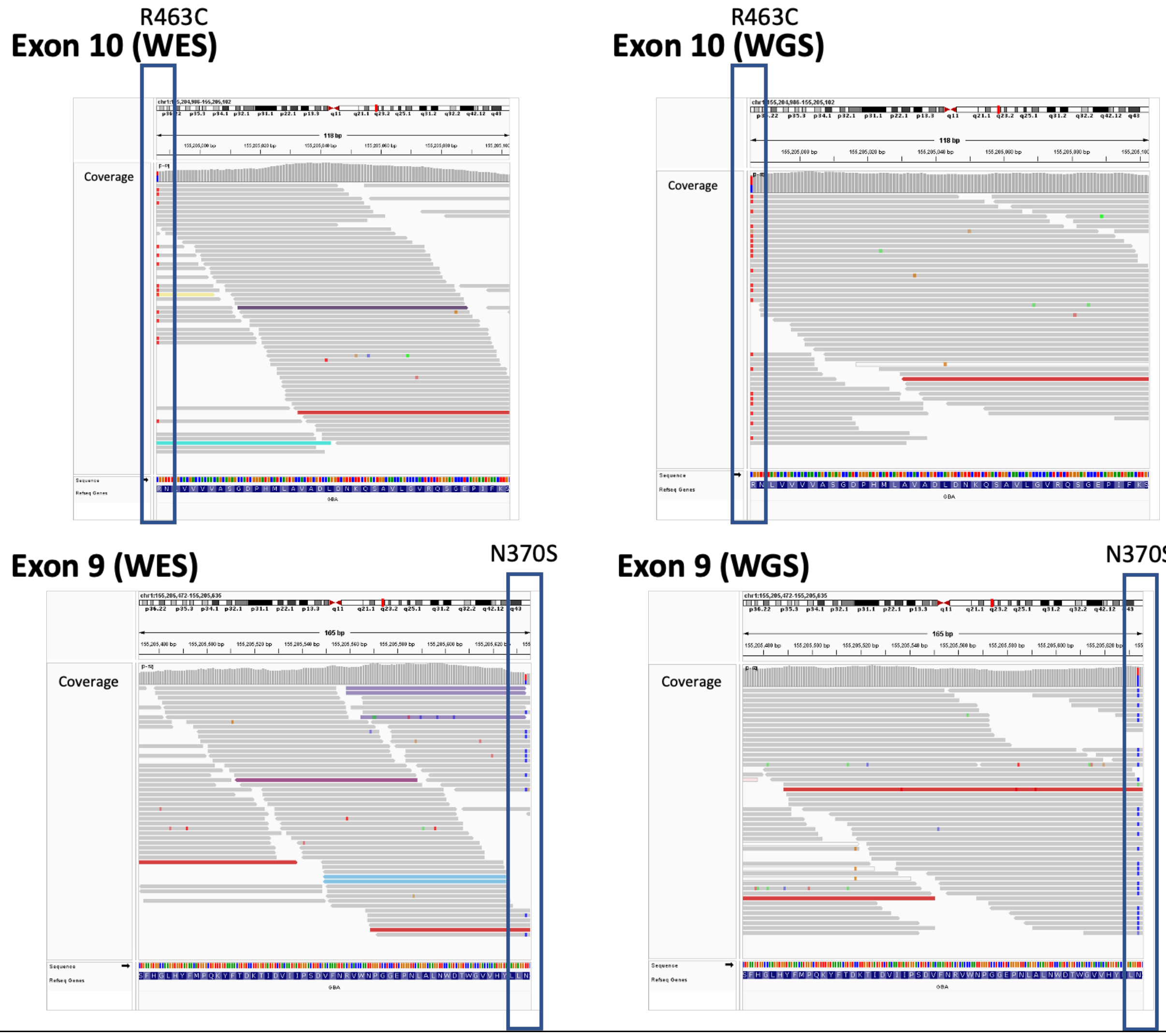

D – Pat_28
Sanger Annotation: R496H/C342Y
Gauchian Call: R496H/WT

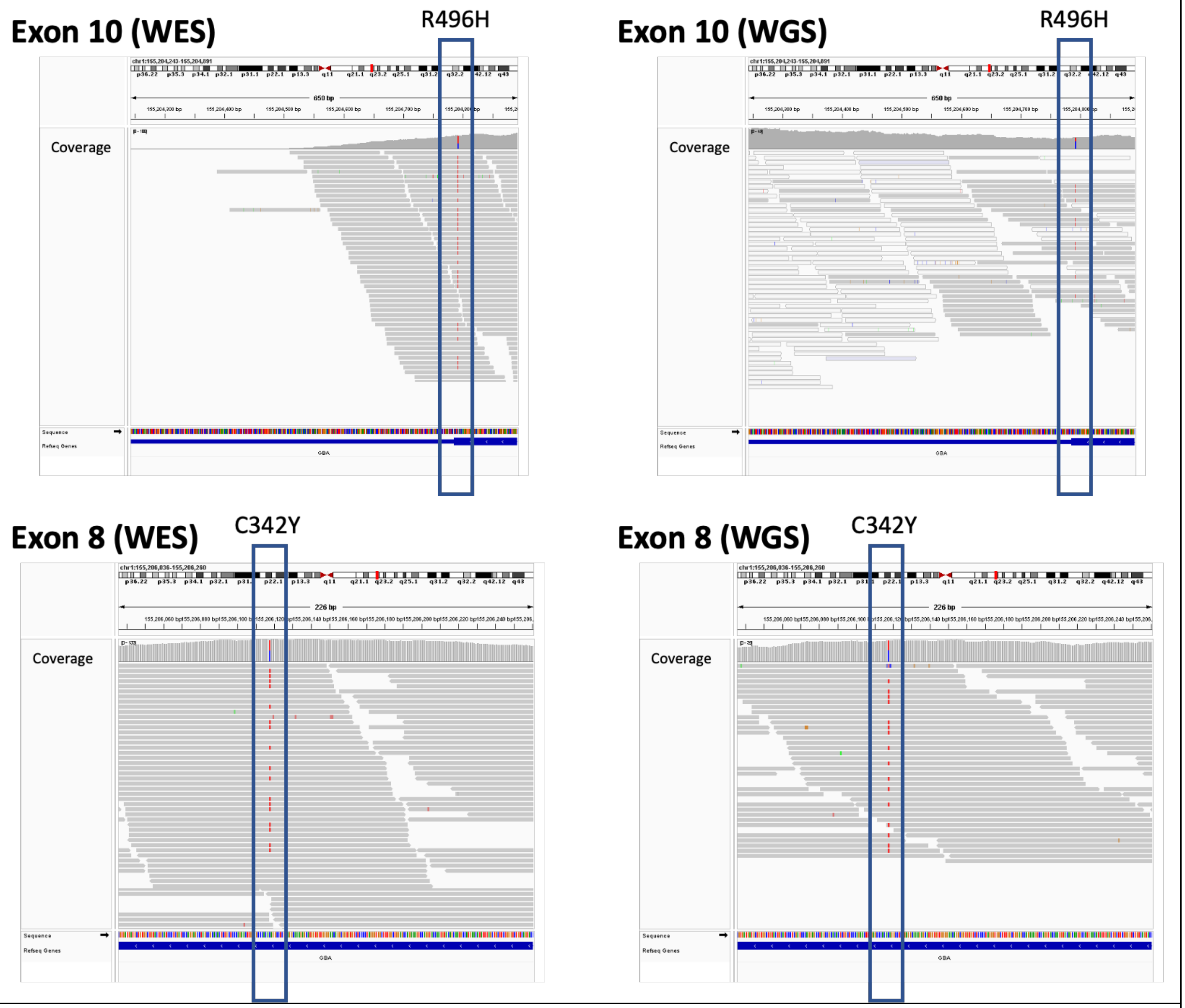

E – Pat_47

Sanger Annotation: N370S/L444P

Gauchian Call: N370S/WT

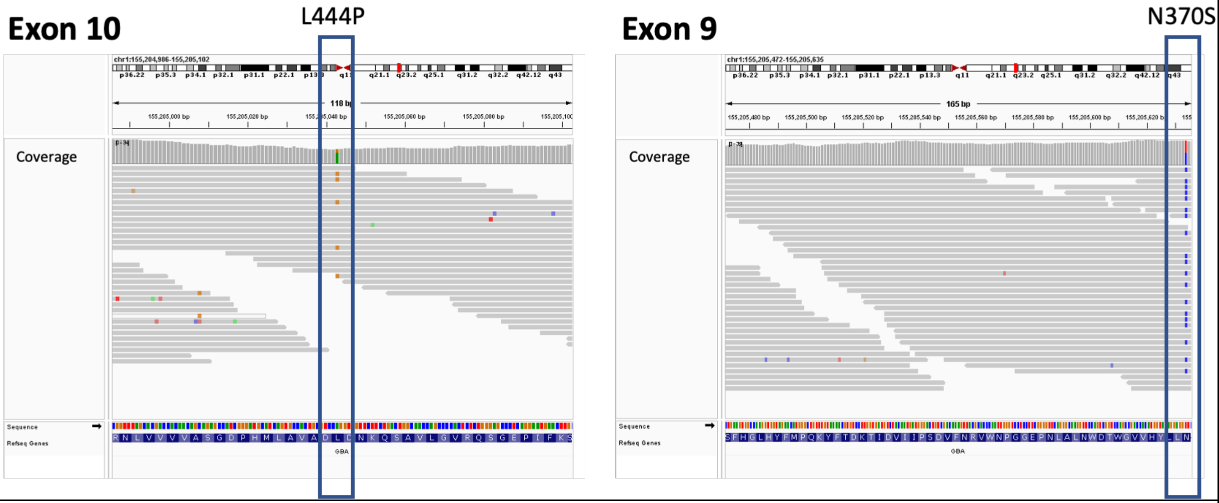

F – Pat_58

Sanger Annotation: N370S/R257*/c203delC
Gauchian Call: N370S/R257*

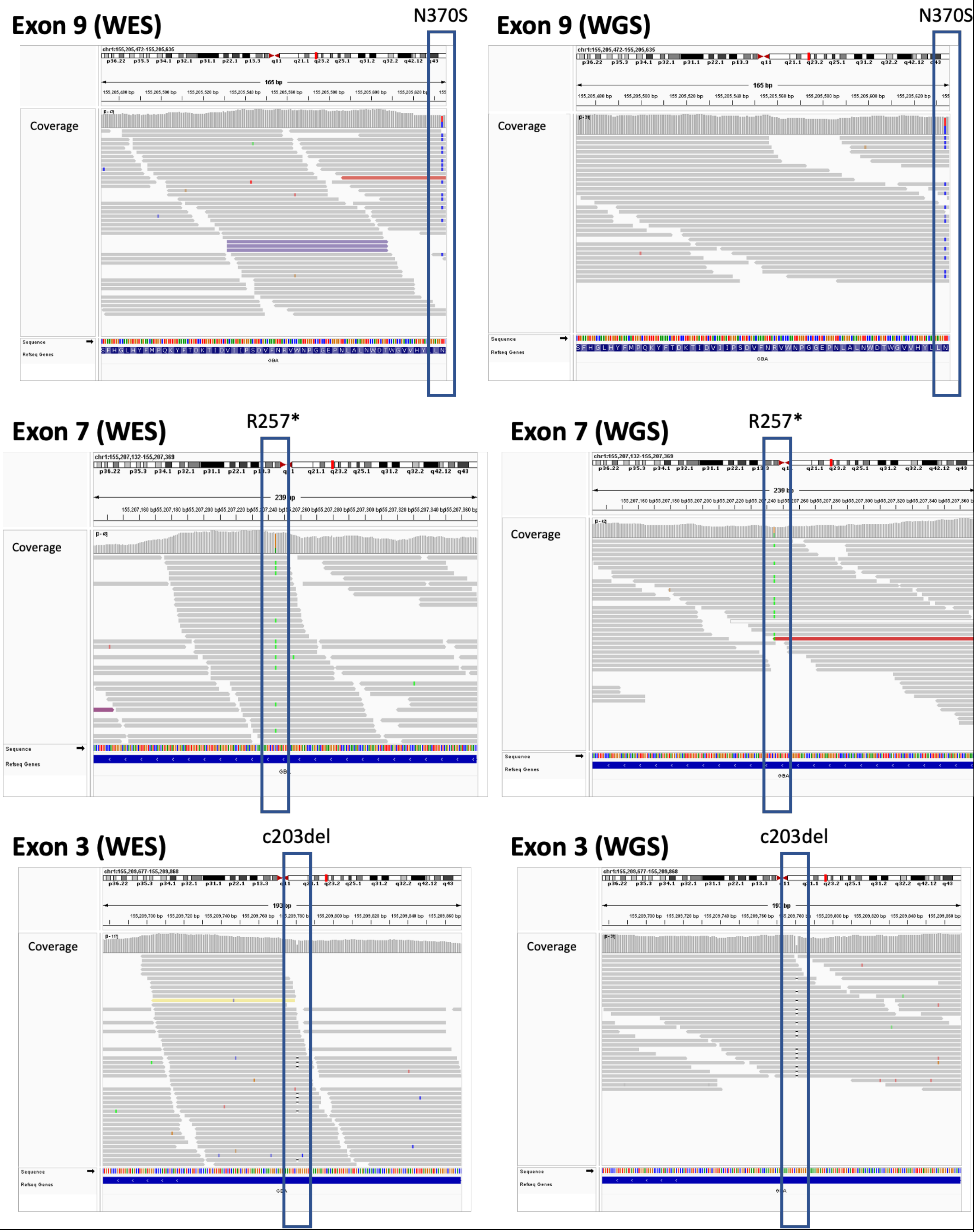

G – Pat_75
Sanger Annotation: R463C/R120W

Gauchian Call: WT/WT

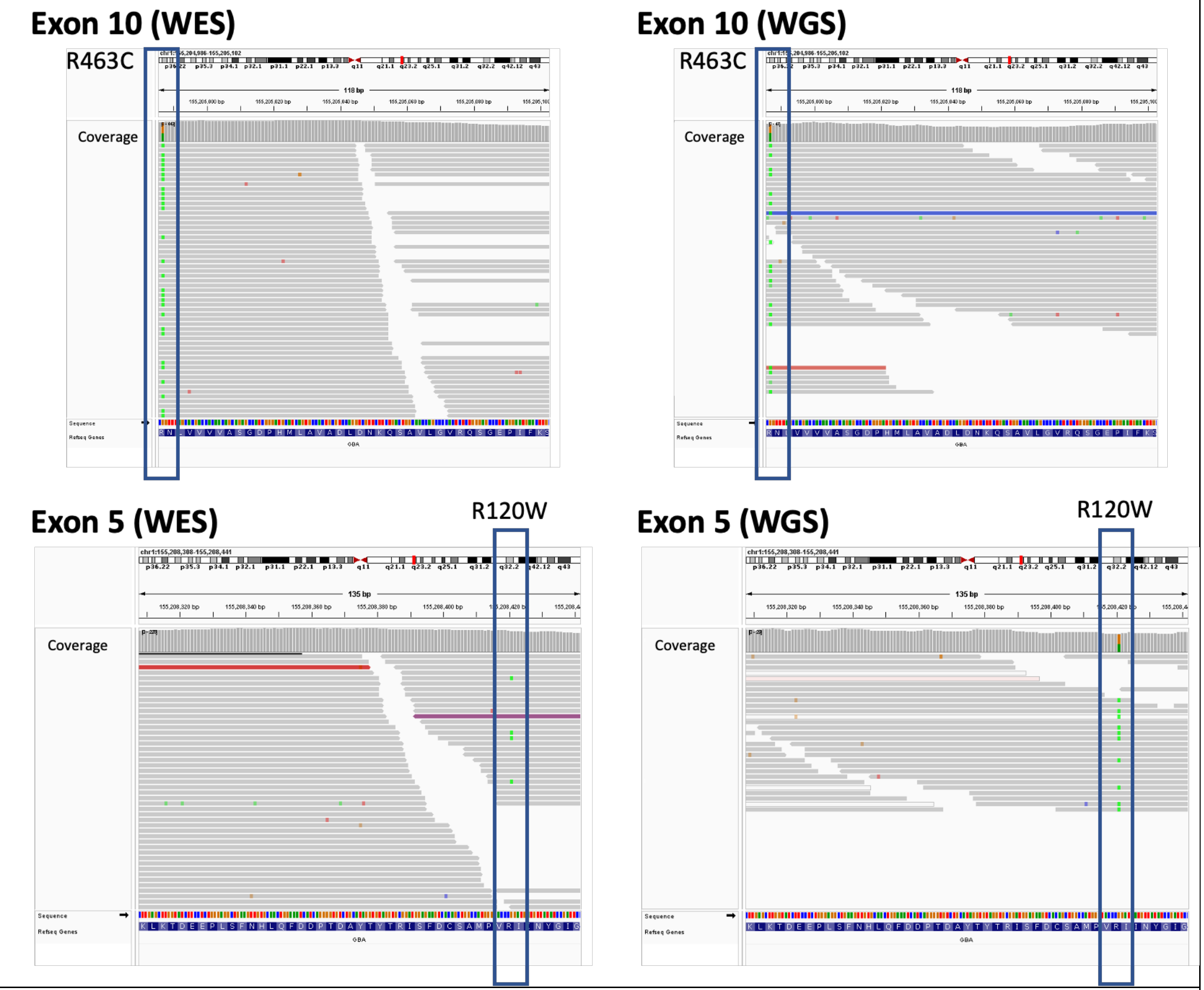

H – Pat_76
Sanger Annotation: N370S/N370S
Gauchian Call: WT/WT

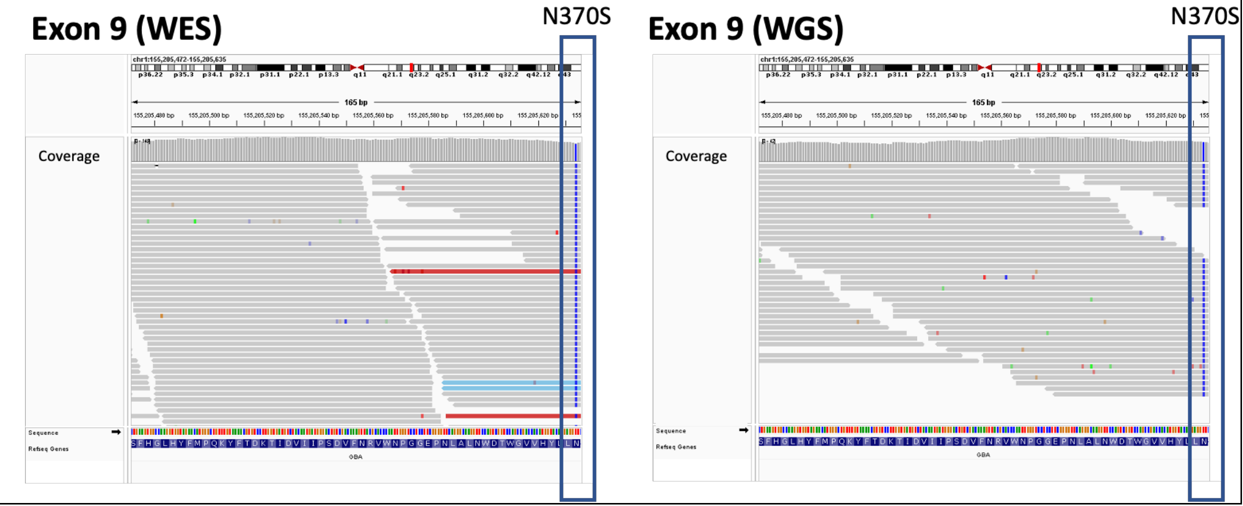

I – Pat_79
Sanger Annotation: L444P/R463C
Gauchian Call: WT/WT

Exon 10 (WGS)

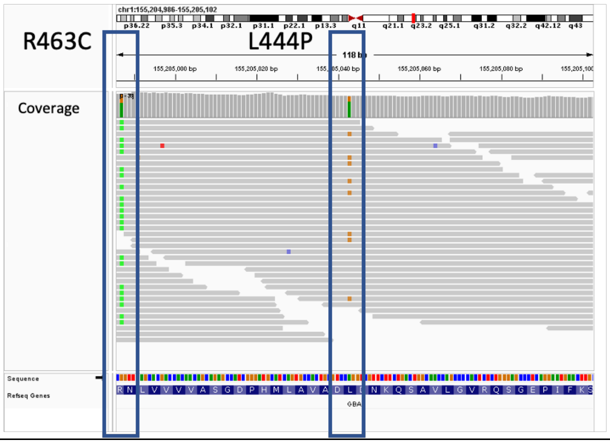

J – Pat_92
Sanger Annotation: D409H/L444P + Rec7
Gauchian Call: D409H/D409H,L444P

Exon 9

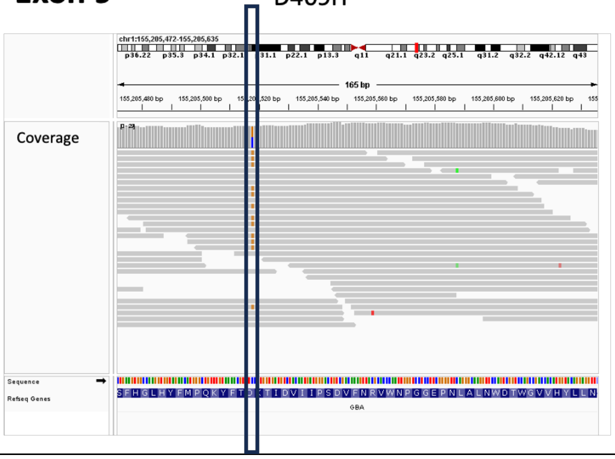

Exon 10

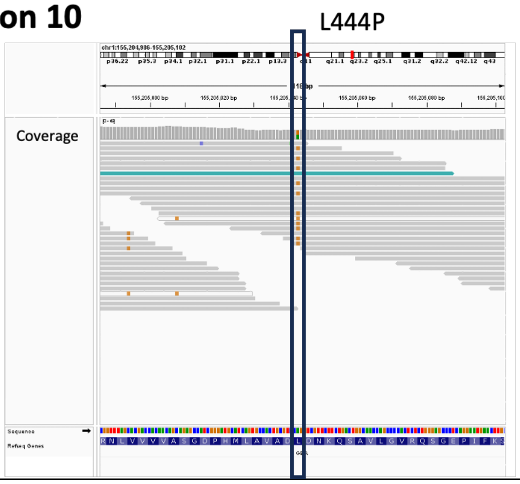

Supplemental Figure 2

Visualization of whole genome sequencing results in patients exhibiting recombinant events.

A – Pat_95 (RecNciI/p.Asn409Ser)

Exon 9 shows a heterozygous mutation for p.Asn409Ser, while exon 10 shows that the variants associated with RecNciI are not explicitly marked and exon 11 shows that the GBA1/GBAP1 SN mismatch is detectable.

Exon 9

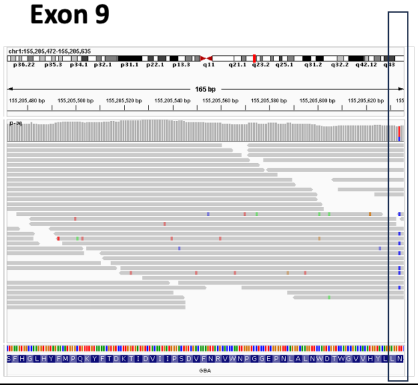

Exon 10

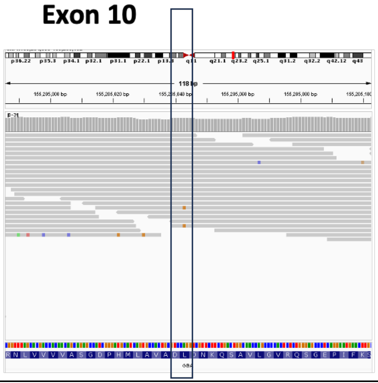

Exon 11

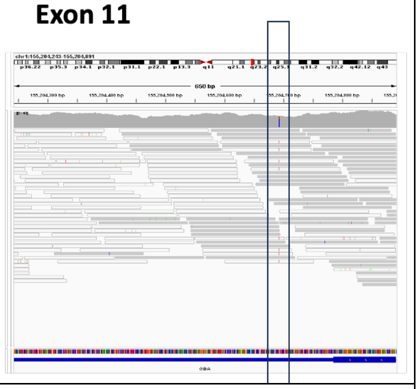

B – Pat_71 (RecNciI/WT)

Exon 10 shows only p.Leu483Pro detectable for the RecNciI variants and the expected mismatches in the 3’-UTR and Intron 9 are undetectable.

Exon 10

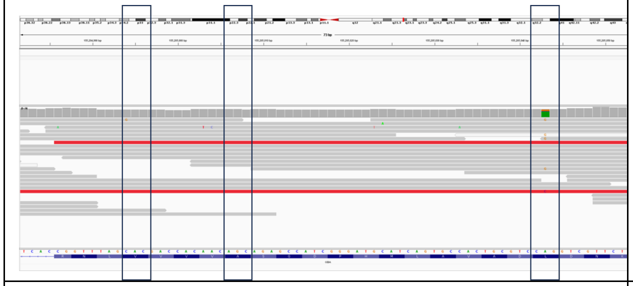

3’UTR

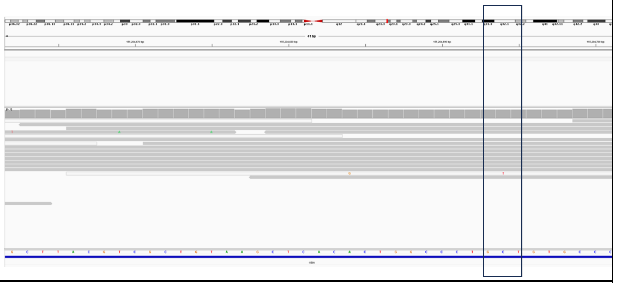

Intron 9

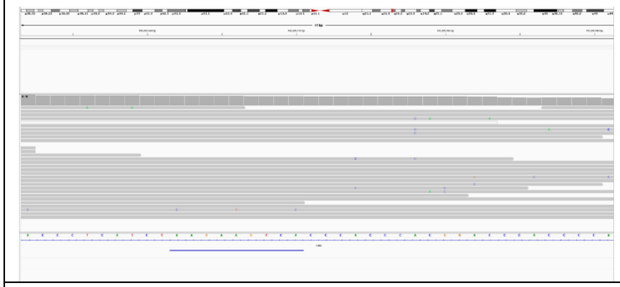

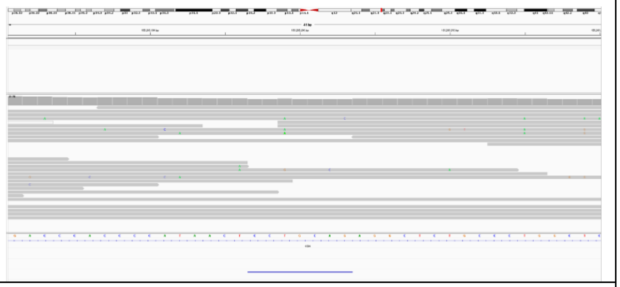

C – Pat_16 (p.Asn409Ser,RecTL+55bpdel)

Exon 9 shows detectable p.Asn409Ser, 55bp deletion but no p.Asp448His. Exon 10 shows that none of the three expected variants are detectable, with the expected mismatches in the 3’UTR also not detectable.

Exon 9

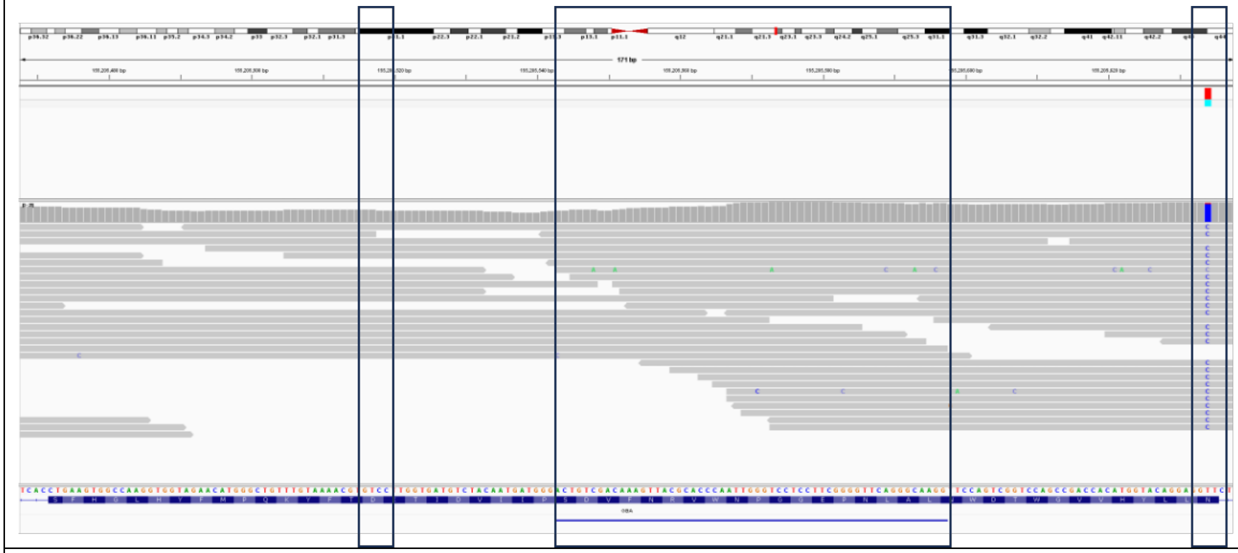

Exon 10

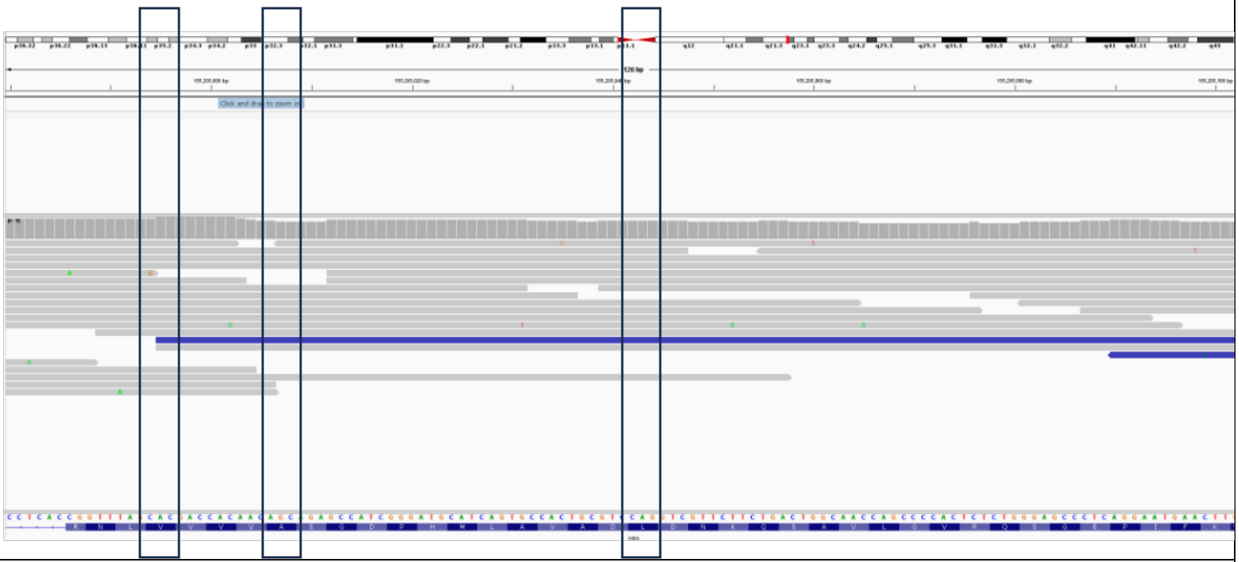

3'UTR

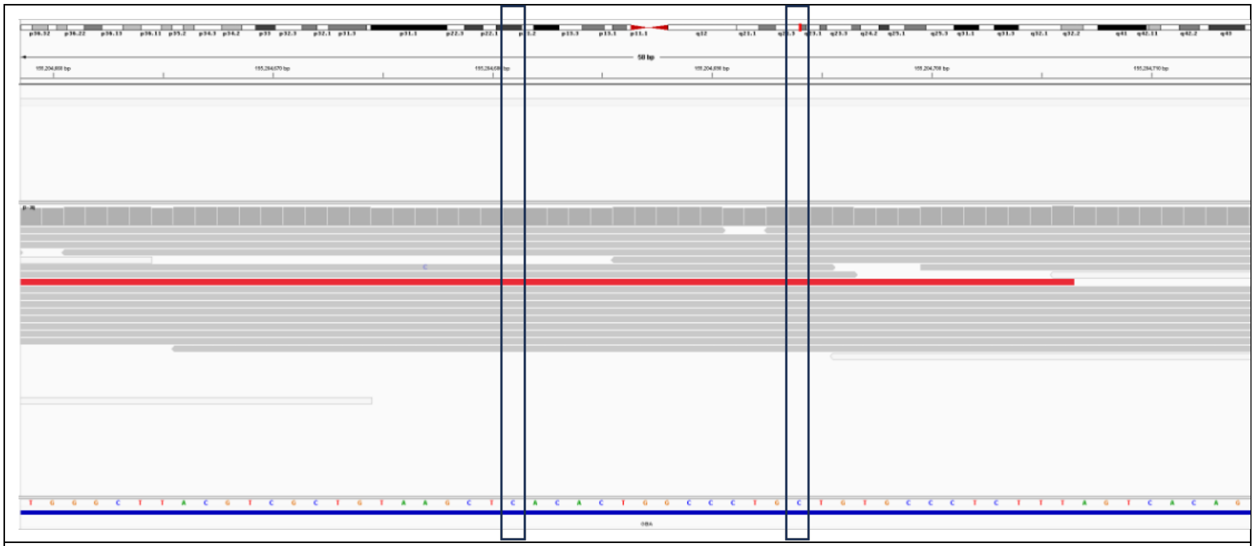

D – Pat_92 (p.Asp448His/p.Leu483Pro, Rec7)
For this *GBA1P* duplication, Gauchian predicted three extra copies (CN=7). The box highlights the possible duplicated area and both missense mutations were detectable.

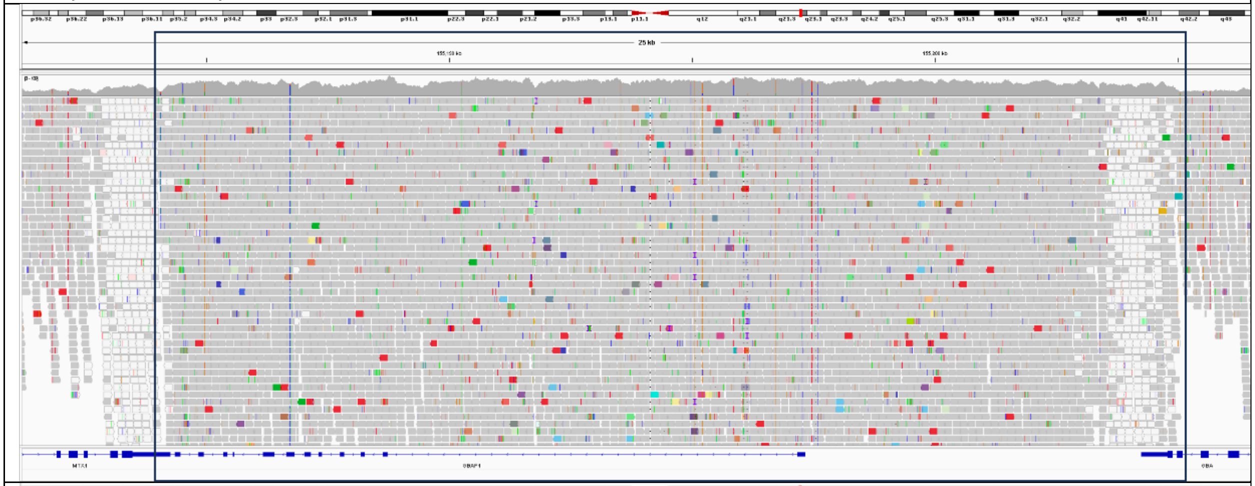

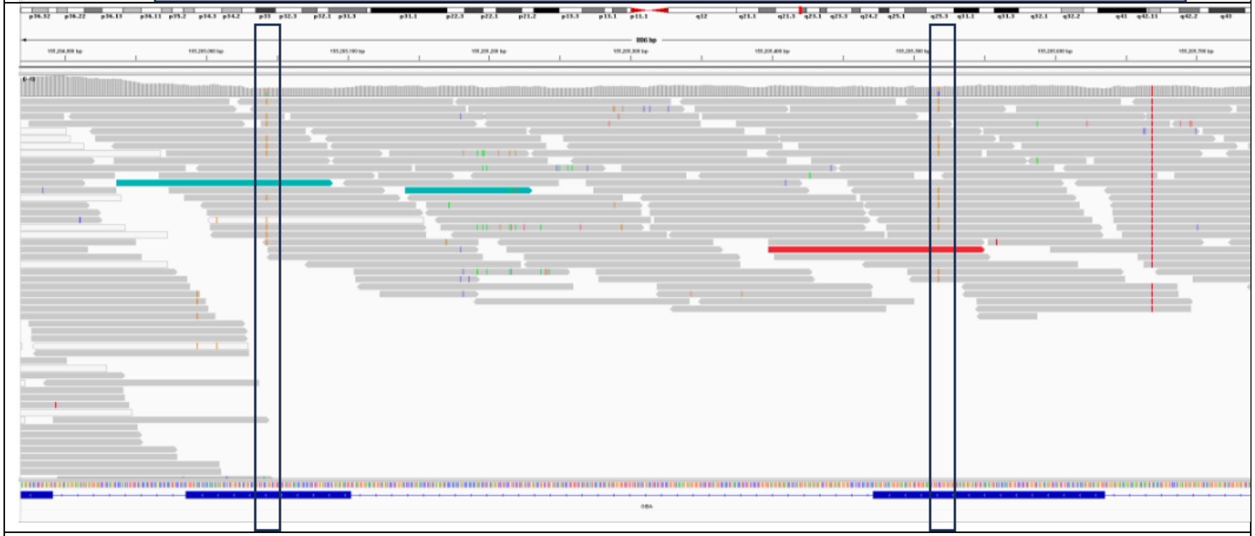

E – Pat_42 (p.Val391Leu/p.Arg398Ter)

For this patient Gauchian predicted two extra copies and a duplication can be detected for *GBAP1*.

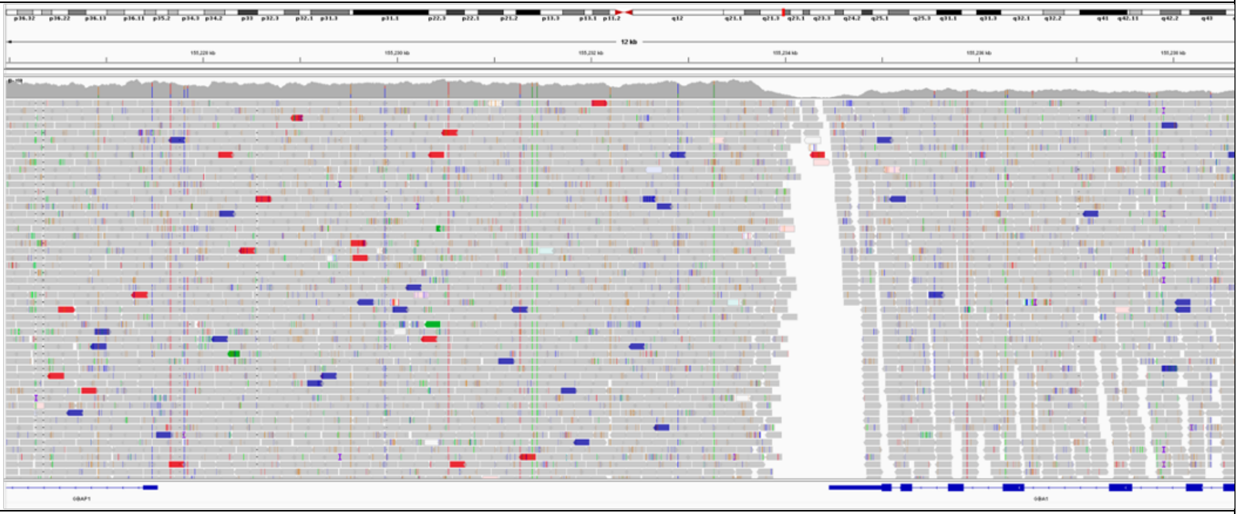

F – Pat_72 (p.Gly241Arg/WT)

For this patient Gauchian predicted one extra copy, which can also be detected for *GBAP1*.

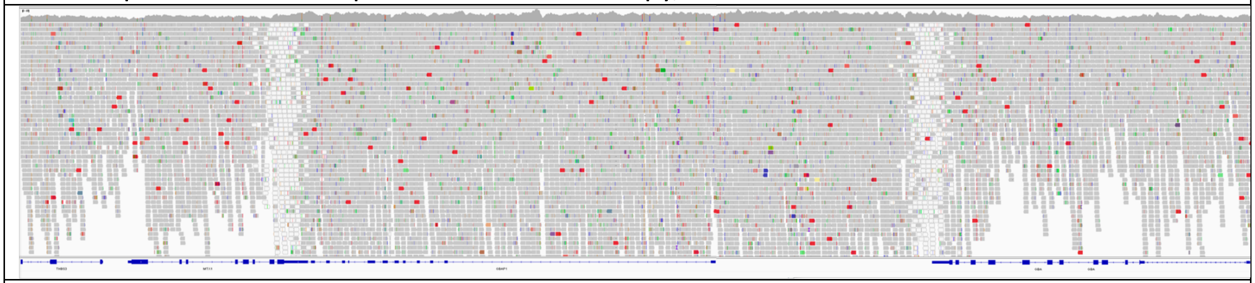

G – Pat_15 (55bpdel/p.Asn409Ser)

The 55bp deletion and the heterozygous p.Asn409Ser mutation in exon 9 are detectable.

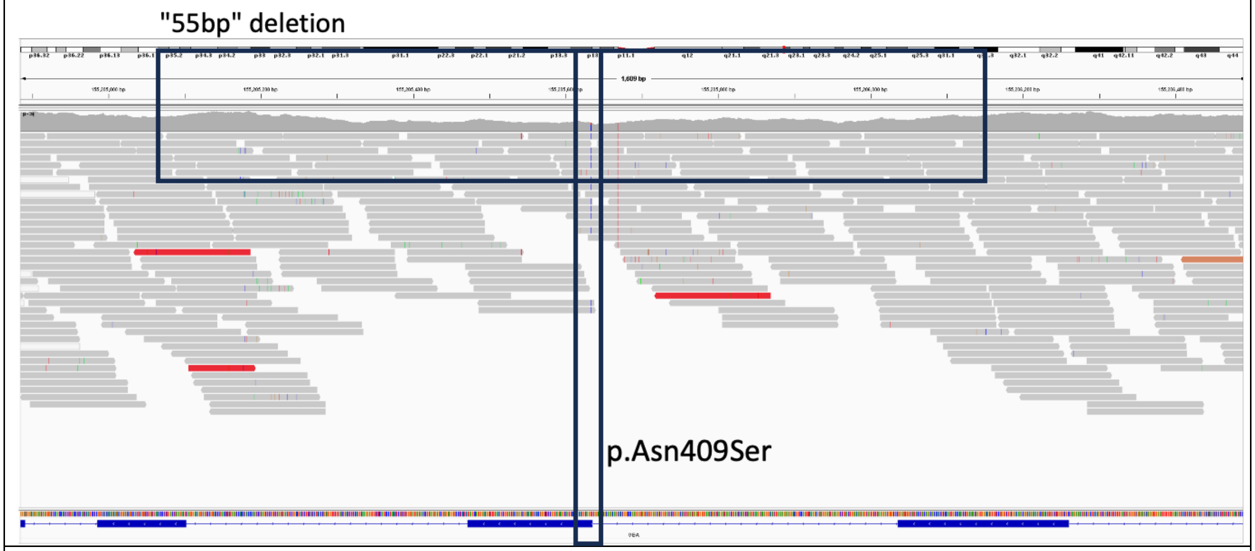

H – Pat_39 (55bpdel/p.Asn409Ser)

The 55bp deletion and the heterozygous p.Asn409Ser mutation in exon 9 are detectable.

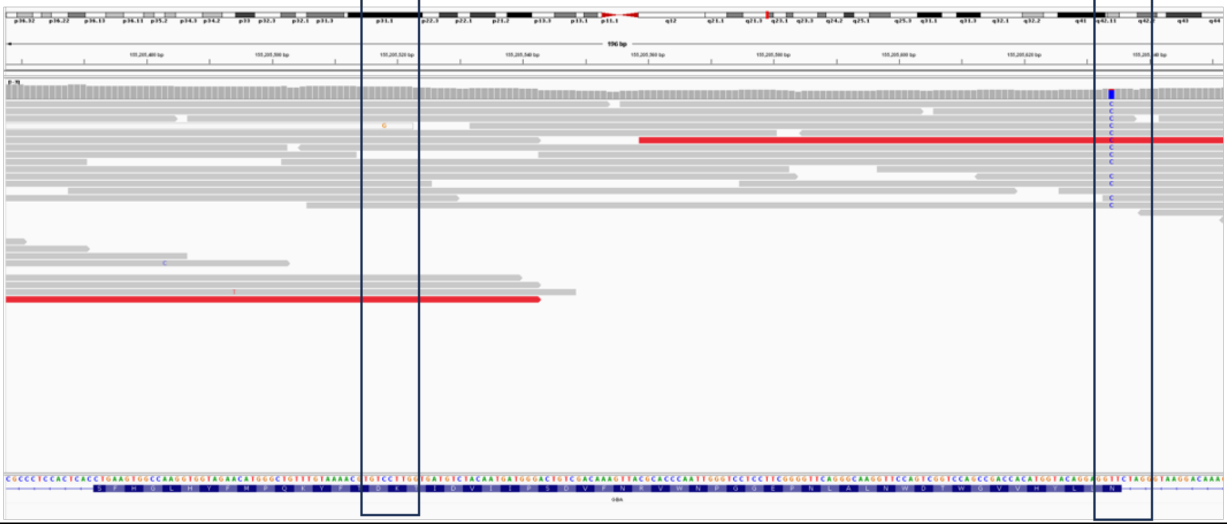

I – Pat_69 (55bpdel/WT)

The 55bp deletion in exon 9 is detectable.

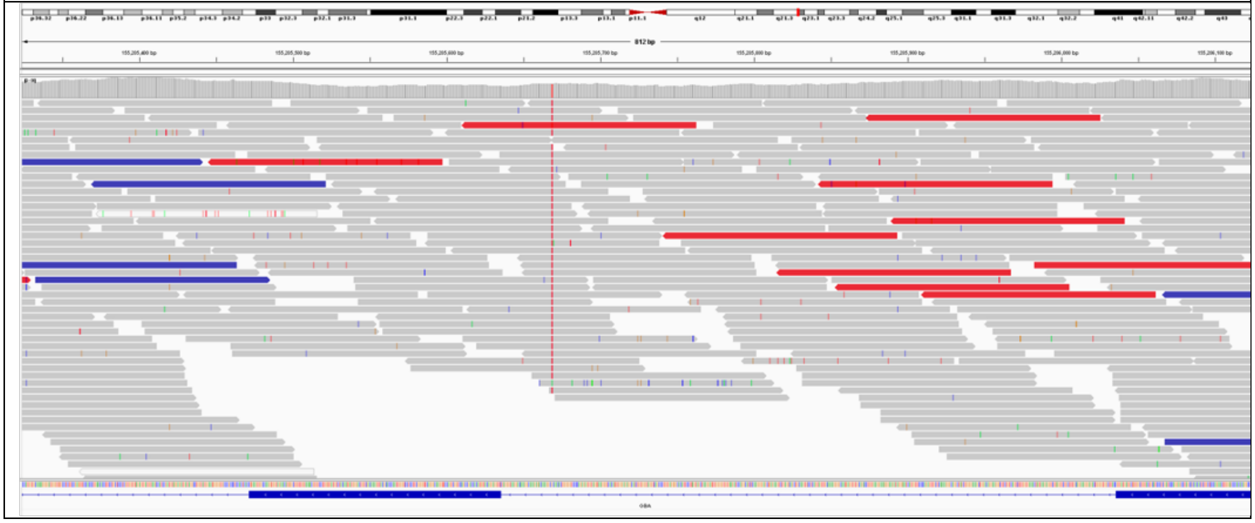

Supplement: Supplement 1 [file media-1.pdf]
